# Likelihood of Neoplasia for Diagnoses Modified by Probability Terms in Canine and Feline Lymph Node Cytology: How Probable Is Probable?

**DOI:** 10.3389/fvets.2018.00246

**Published:** 2018-10-09

**Authors:** Mary M. Christopher, Chieh-Ko Ku

**Affiliations:** Department of Pathology, Microbiology and Immunology, School of Veterinary Medicine, University of California, Davis, Davis, CA, United States

**Keywords:** biopsy, fine-needle aspirate, lymphoma, metastatic neoplasia, small animal

## Abstract

**Background:** Descriptive probability modifiers are used often to convey the uncertainty of a pathology diagnosis, but they also contribute to ambiguity in communication between pathologists and clinicians.

**Objectives:** Our goal was to determine the frequency and use of probability modifiers in canine and feline lymph node cytology diagnoses, and to determine the actual likelihood of neoplasia for diagnoses with and without modifiers, based on the histologic outcome.

**Methods:** Canine and feline lymph node cytology and histology reports over an 11-year period (2001–2011; *n* = 367) were evaluated retrospectively. Diagnoses were categorized as neoplastic/malignant (lymphoma, metastatic) or non-neoplastic/benign. The frequency and type of modifier, and the sensitivity, specificity, and predictive values for neoplasia were determined for modified and unmodified diagnoses using histology as the gold standard.

**Results:** Ninety-one of 367 (24.8%) cytology diagnoses were modified by probability terms, including 25/204 (12.2%) diagnoses of non-neoplastic lesions and 66/163 (40.5%) diagnoses of neoplasia. In addition, 26 unmodified diagnoses of neoplasia were followed by a probability phrase indicating specific tumor type. Based on the histologic outcome, modified diagnoses had higher sensitivity (87.3%, confidence interval [CI] 75.5, 94.7%) but lower specificity (50.0%, CI 32.9, 67.1%) for neoplasia than did unmodified diagnoses (60.6 and 100%, respectively; *P* < 0.0001, Chi square). Modified phrases indicating the probability of a specific tumor type were accurate in 22/26 (84.6%) cases. Positive predictive values for neoplasia were 100% (CI 96.2, 100%) for unmodified and 72.7% (CI 60.4, 83.0%) for modified diagnoses. Negative predictive values were 65.9% (CI 58.5, 72.8%) for unmodified and 72.0% (CI 60.4, 83.0%) for modified diagnoses. No significant difference was found in the likelihood of neoplasia for individual terms used to modify a cytologic diagnosis except for “cannot rule out” (*P* = 0.0368).

**Conclusions:** Most modified diagnoses of cancer in canine and feline lymph node cytology have a 60–83% likelihood of neoplasia based on histologic outcome, compared with 96–100% for unmodified diagnoses. Non-neoplastic lesions, regardless of modifiers, have a 12–49% likelihood of neoplasia. A limited number of risk categories based on these likelihoods may be a more effective and accurate way to communicate the risk of malignancy in lymph node cytology.

## Introduction

A written cytology report is the principal means by which diagnostic results are communicated from the clinical pathologist to the clinician (1, 2). Uncertainty is inherent in many cytology and pathology diagnoses, with certainty being affected by the quality of sample, type of lesion, pathologist experience, and availability of patient information (2–4). Pathologists, like other medical professionals (5), often convey the uncertainty or probability of a diagnosis using descriptive terms, such as “probable,” “suggestive,” and “compatible with” (3, 6–9). However, there is wide variation and overlap in how such terms are interpreted by pathologists and by clinicians (3, 4, 6–9). This can contribute to miscommunication and has been shown to affect clinical management and decision-making, including the decision to euthanize (10, 11). Defined categories of diagnostic probability based on the evidence-based likelihood of disease would provide more meaningful results than subjective terminology.

In medical cytopathology, standardized reporting categories have been developed for gynecologic, thyroid, pancreaticobiliary, urinary, and salivary gland samples to improve the uniformity of pathologist communication with clinicians, radiologists, and patients, and to facilitate cytologic-histologic correlation, data-sharing, and research (12–14). Each diagnostic category (which sometimes include probability terms) is associated with a specific risk of malignancy and with a recommendation for management. For example, a diagnosis of “suspicious for malignancy” in a thyroid aspirate conveys a 50–75% risk of malignancy, for which the recommended management is thyroidectomy or surgical lobectomy (13). Risk categories can be institutionally validated and are routinely updated in response to outcomes-based research (13–16). A similar system has been applied to the cytologic diagnosis of metastatic mast cell disease in dogs, in which diagnoses of “possible metastasis” and “probable metastasis” correspond to specific cytologic findings that correlate with tumor grade and outcome (17). However, for most cytopathology diagnoses in animals, it is unknown how subjective expressions of probability correspond to the actual likelihood or risk of malignancy or disease.

In past studies, veterinary clinical pathologists and clinicians were asked to hypothetically estimate the percentage likelihood implied by different probability modifiers (3, 11). To our knowledge, studies to determine the actual accuracy and outcomes of modified versus unmodified diagnoses, based on a gold standard, have not been done. The purpose of this study was to determine the frequency and use of probability modifiers in canine and feline lymph node cytology reports in a tertiary care veterinary teaching hospital, and to determine the diagnostic accuracy and actual likelihood of neoplasia for diagnoses with and without modifiers, based on histologic outcomes. Lymph nodes are frequently examined by cytologic methods in dogs and cats, and neoplasia in a lymph node is generally always malignant. The results of this study will inform the use and interpretation of probability terms by pathologists and clinicians and could form the basis for future standardized reporting categories that improve the effectiveness and accuracy of communicating the likelihood of neoplasia in lymph node cytology specimens.

## Materials and methods

### Study design and population

We used an existing database of lymph node cytology and histology diagnoses from dogs and cats, which we analyzed previously for cytology-histology concordance (18). In that study, probability modifiers used with cytology (and histology) diagnoses were ignored: any diagnosis where neoplasia was reported as a possibility was classified as neoplastic. For the present study, modified and unmodified diagnoses were compared separately for their concordance with histologic diagnoses. The diagnostic accuracy and actual likelihood of neoplasia were then determined, using histology as the gold standard.

Database retrieval and inclusion and exclusion criteria were reported previously (18). Briefly, 367 cases (296 dogs, 71 cats), each consisting of a single paired cytology-histology result, were evaluated at the Veterinary Medical Teaching Hospital at the University of California–Davis over an 11-year period (January 2001 through December 2011). Cytology specimens were obtained by fine-needle aspiration or impression smears and stained with Wright-Giemsa. Histology specimens were obtained using needle core or surgical biopsy (or necropsy in 4 cases), and were fixed, sectioned, and stained routinely using H&E. Most cases of lymphoma were subclassified as T-cell or B-cell based on immunohistochemical staining. Board-certified duty pathologists finalized all reports.

### Classification of diagnoses and probability modifiers

Cytology and histology diagnoses were categorized as non-neoplastic (benign) or neoplastic (malignant). Benign diagnoses included normal, reactive/hyperplastic, and inflammatory. Malignant diagnoses included lymphoma and metastatic neoplasia (mast cell tumor, histiocytic sarcoma, other hemic neoplasia, carcinoma, sarcoma, melanoma, or unspecified neoplasia). When both benign and malignant processes were reported in the same node, the final diagnosis was categorized as neoplasia. Cytology and histology diagnoses were considered to be in complete agreement (neoplastic vs. non-neoplastic), partial agreement (agreed on neoplastic vs. non-neoplastic, but disagreed on the type of non-neoplastic process or tumor), or disagreement.

The type and frequency of probability modifiers applied to any portion of the cytology diagnosis were recorded; microscopic descriptions and comments sections were not analyzed. Modifiers used solely to qualify the specific tumor type for an already unmodified diagnosis of neoplasia (e.g., carcinoma, most likely transitional cell carcinoma) were recorded separately. Modified and unmodified cytology diagnoses were categorized as true-negative, true-positive, false-positive, or false-negative based on the histologic diagnosis (neoplastic vs. non-neoplastic).

### Statistical analysis

Sensitivity, specificity, and overall diagnostic accuracy (and corresponding 95% confidence intervals [CIs]) were calculated for modified and unmodified diagnoses. The likelihood of neoplasia with a cytologic diagnosis of neoplasia (positive predictive value, PPV) was calculated as the number of true-positive results (complete and partial agreement combined) divided by the number of true-positive and false-positive results. The likelihood of a non-neoplastic lesion with a cytologic diagnosis of a non-neoplastic lesion (negative predictive value, NPV) was calculated as the number of true-negative results (complete and partial agreement combined) divided by the number of true-negative and false-negative results. The overall proportion of accurate diagnoses (true positive and true negative) and the PPV and NPV also were calculated for specific modifiers. Differences in results based on modifier, species, anatomic site of the lymph node, and type of neoplasm or benign process were assessed using the Chi-square test (JMP v. 12.0.1. SAS Institute, Cary, NC, USA). All statistical tests were two-sided at a significance level of 0.05.

## Results

**Figure 1:**
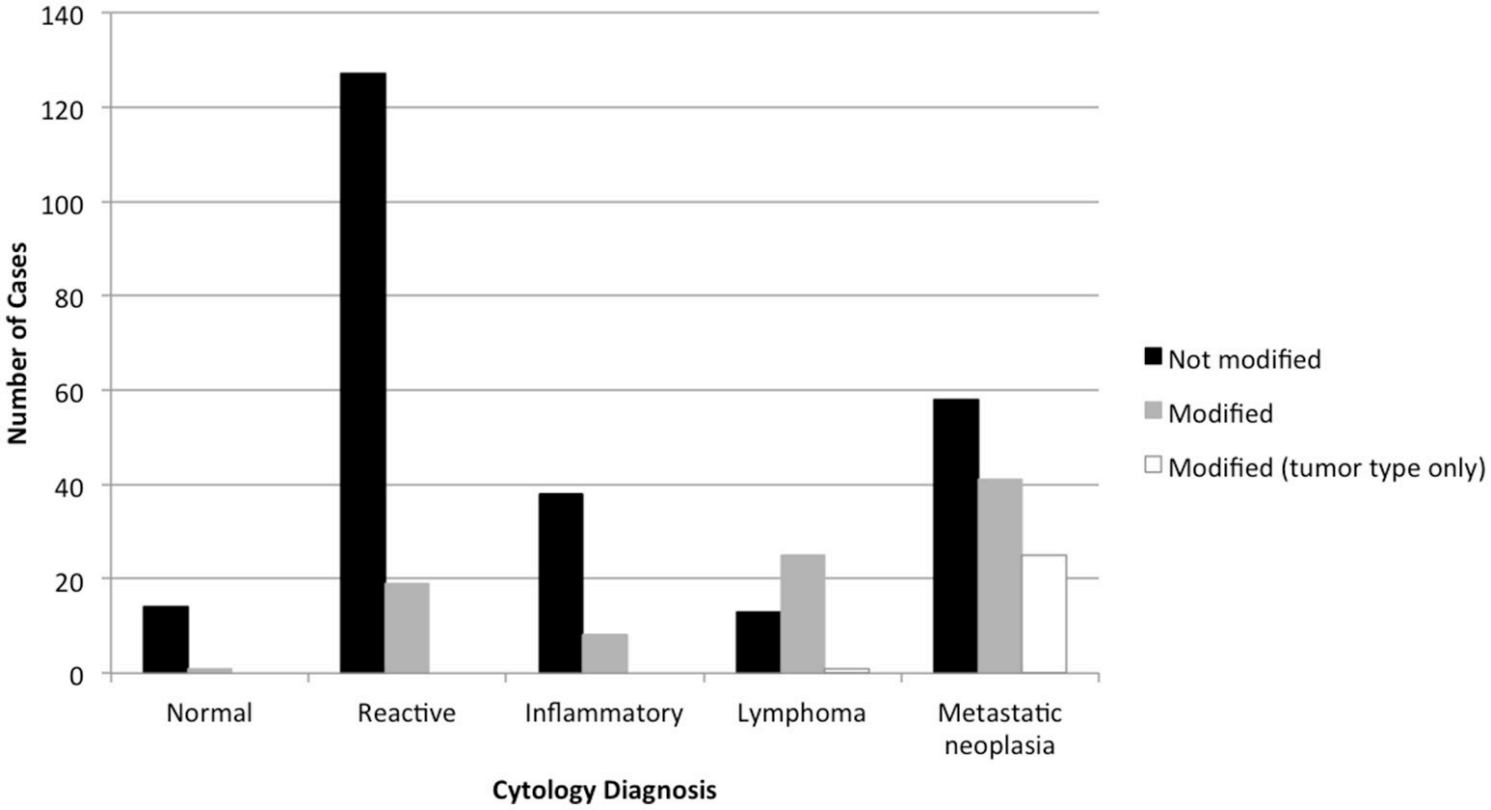
Diagnoses modified by probability terms in lymph node cytology reports, based on the primary cytologic diagnosis. Diagnoses of neoplasia were significantly more likely to be modified than non-neoplastic processes, and diagnoses of lymphoma were significantly more likely to be modified than metastatic neoplasia.

Ninety-one of 367 (24.8%) lymph node cytology diagnoses were modified by probability terms while 276/367 (75.2%) were unmodified (Table 1). In 3 reports, both non-neoplastic and neoplastic diagnoses were modified (i.e., probable reactive lymphoid hyperplasia, cannot rule out lymphoma). Cytologic diagnoses of neoplasia were 3.3X more likely to be modified than non-neoplastic lesions (*P* < 0.0001). Diagnoses of lymphoma were twice as likely to be modified (25/39, 64.1%) as diagnoses of metastatic neoplasia (39/122, 32.0%) (*P* = 0.0004) (Figure 1); there was no significant difference in the rate of modifier use for diagnoses of lymphoid reactivity and inflammation. Significantly fewer cytologic diagnoses of neoplasia (58/145, 40.0%) and significantly more diagnoses of non-neoplastic lesions (19/151, 12.5%) were modified in dogs as compared with cats (neoplastic 8/18, 44.4%; non-neoplastic 6/53, 11.3%) (*P* = 0.0033). No significant difference was found in the proportion of modified and unmodified diagnoses based on lymph node site.

**Table 1:** Frequency of diagnoses modified by probability terms in lymph node cytology reports from dogs and cats.

**Cytology diagnosis**	**Unmodified**	**Modified**	**Total (% Modified)**
Non-neoplastic (*n* = 204)	179	25	25/204 (12.2%)
Neoplastic (*n* = 163)	97	66^*^	66/163 (40.5%)
Total	276	91	91/367 (24.8%)

* *Includes 3 reports in which diagnoses of both neoplastic and non-neoplastic lesions were modified; does not include 26 modified diagnoses of specific tumor type that followed an unmodified diagnosis of neoplasia*.

Unmodified (vs. modified) diagnoses of neoplasia were significantly more likely to be in complete agreement with the histologic diagnosis, while unmodified diagnoses of non-neoplastic lesions were significantly more likely to be in disagreement with the histologic diagnosis (*P* < 0.0001; Table 2). Three unmodified diagnoses of metastatic carcinoma by cytology were in disagreement with the histologic diagnosis of reactive hyperplasia, however, because cytologic evidence of malignancy was definitive and the primary tumor was histologically confirmed as carcinoma, these samples were considered as false-negative biopsy results and were not included in the calculation of diagnostic accuracy. The overall prevalence of neoplasia based on the histologic diagnosis was 39.5% (145/367).

**Table 2:** Level of agreement with histology of unmodified and modified cytology diagnoses.

**Agreement with histology**	**Unmodified neoplasia**	**Modified neoplasia**	**Unmodified non-neoplastic**	**Modified non-neoplastic**	**Total (%)**
Complete	86 (88.6%)	42 (63.6%)	92 (51.4%)	15 (60.0%)	235 (64.0%)
Partial	8 (8.2%)	6 (9.1%)	26 (14.5%)	3 (12.0%)	43 (11.9%)
Disagreement	3 (3.0%)^*^	18 (27.2%)	61 (34.0%)	7 (28.0%)	89 (24.2%)
Total	97	66	179	25	367

* *Carcinoma in cytology, reactive hyperplasia in histology*.

The diagnostic accuracy of modified diagnoses was 5.2% lower than that of unmodified diagnoses (Table 3). The sensitivity of modified diagnoses was significantly higher (fewer false negatives) but specificity was significantly lower (more false positives) compared with unmodified diagnoses (*P* < 0.0001). For dogs considered separately (*n* = 293), modified diagnoses had a sensitivity of 95.4% (CI 84.5, 99.4%) compared with 67.2% (CI 58.2, 75.3%) for unmodified diagnoses. Specificity in dogs was similar to that of all samples combined (100% for unmodified, 51.5% for modified). In cats (*n* = 71), modified diagnoses had a sensitivity of 54.5% (CI 23.4, 83.3%) compared with 33.3% (CI 17.3, 52.8%) for unmodified diagnoses. Specificity in cats was 100% for unmodified diagnoses, but could not be accurately calculated for modified diagnoses because of the low number of samples. The likelihood of neoplasia with a cytologic diagnosis of neoplasia (PPV) was 100% (94/94; CI 96.2, 100%) for unmodified and 72.7% (48/66; CI 60.4, 83.0%) for modified diagnoses. The likelihood of a non-neoplastic lesion with a cytologic diagnosis of a non-neoplastic lesion (NPV) was 65.9% (118/179; CI 58.5, 72.8%) for unmodified and 72.0% (18/25; CI 50.6, 87.9%) for modified diagnoses.

**Table 3:** Sensitivity, specificity, and accuracy of lymph node cytology for the diagnosis of neoplasia when diagnoses are unmodified or modified by probability terms.

**Cytologic diagnosis**	**Histologic diagnosis**		
**Unmodified**	**Non-neoplastic**	**Neoplastic**	**Total**
Non-neoplastic	118 (TN)	61 (FN)	179
Neoplastic	0 (FP)^*^	94 (TP)	94
Total	118	155	273
	Specificity = 100% (CI 96.9, 100%)	Sensitivity = 60.6% (CI 52.5, 68.4%)	Accuracy = 77.7% (CI 72.2, 82.5%)
**Modified**	**Non-neoplastic**	**Neoplastic**	**Total**
Non-neoplastic	18 (TN)	7 (FN)	25
Neoplastic	18 (FP)	48 (TP)^†^	66
Total	36	55	91
	Specificity = 50.0% (CI 32.9, 67.1%)	Sensitivity = 87.3% (CI 75.5, 94.7%)	Accuracy = 72.5% (CI 62.2, 81.4%)

* *Does not include 3 samples where metastatic carcinoma was diagnosed in cytology but not in biopsy specimens*.

† *Does not include 26 cases where modifiers were applied only to the specific tumor type*.

*TN indicates true negative; FN, false negative; FP, false positive; TP, true positive; CI confidence interval*.

**Figure 2:**
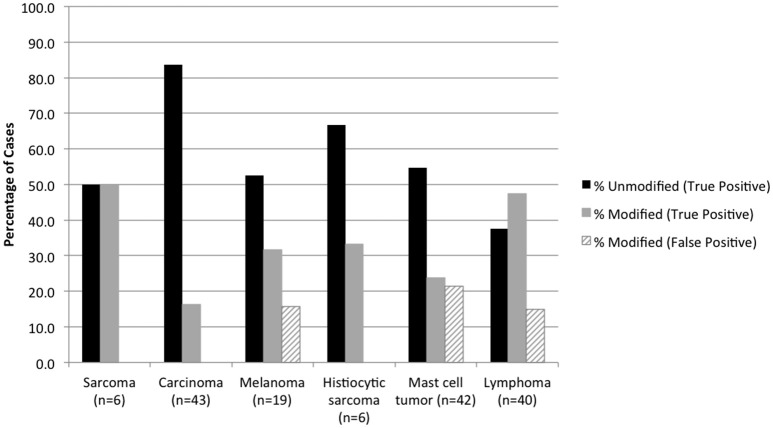
Percentage of true positive and false positive diagnoses of neoplasia in modified and unmodified cytology reports, based on the histologic diagnosis.

**Figure 3:**
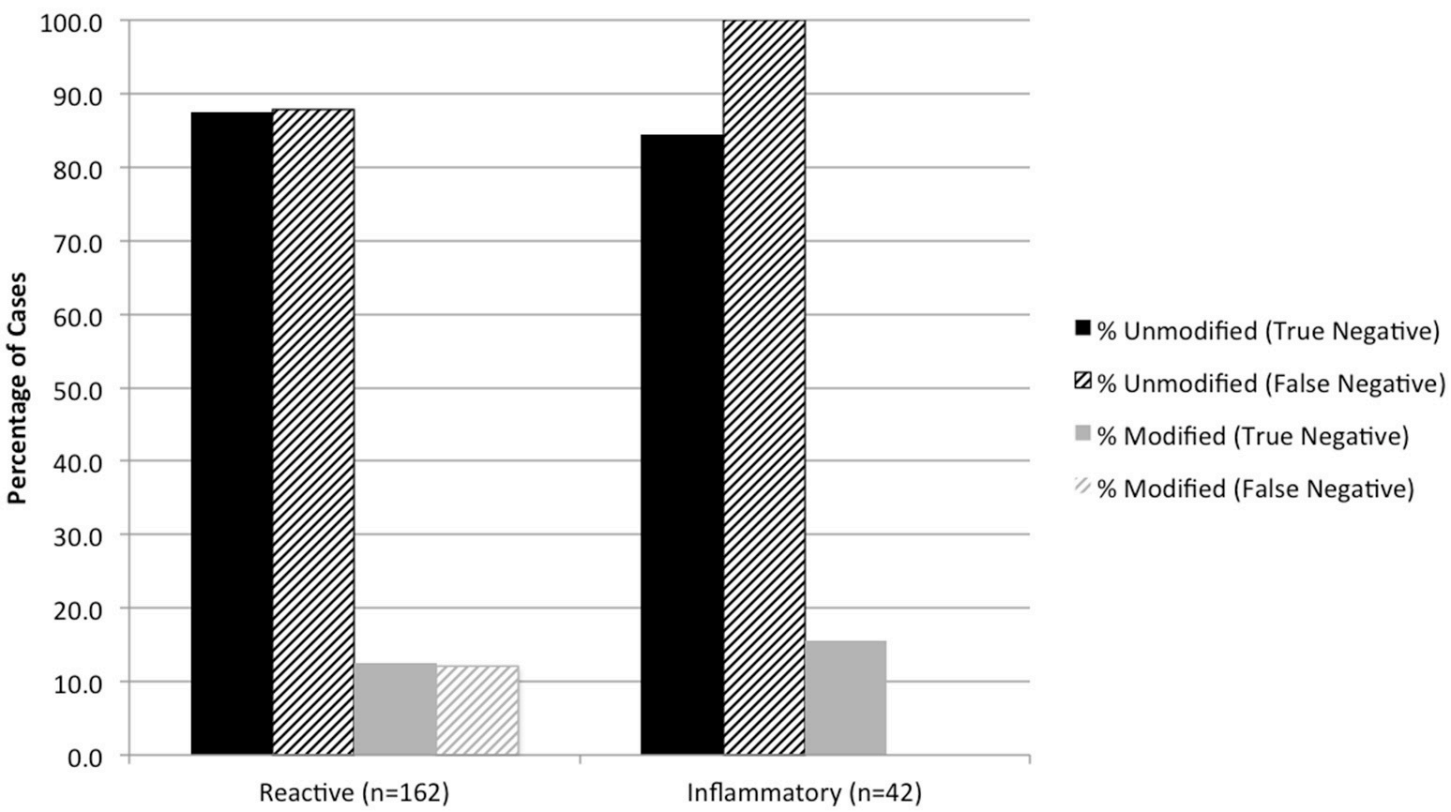
Percentage of true negative and false negative diagnoses of reactive and inflammatory lesions in modified and unmodified cytology reports, based on the histologic diagnosis.

Cytologic diagnoses of lymph nodes that were histologically confirmed as having lymphoma and sarcoma were significantly more likely to be modified than carcinoma and other metastatic neoplasms (*P* < 0.0001) (Figure 2). False-positive cytology diagnoses were always modified, and comprised 3 of 19 (15.8%) metastatic melanomas, 9 of 42 (21.4%) mast cell tumors, and 6 of 40 (15.0%) lymphomas. Two of the false-positive diagnoses were from mesenteric nodes in cats (1 mast cell tumor, 1 lymphoma); the remaining 16 false-positive diagnoses were from popliteal (5 mast cell tumors, 1 lymphoma), mandibular (3 melanomas, 3 lymphomas, 2 mast cell tumors), and prescapular (1 lymphoma, 1 mast cell tumor) nodes in dogs. There was no significant difference in the proportion of modified reactive and inflammatory nodes (based on the histologic diagnosis) or in the proportion of modified true-negative and false-negative diagnoses (Figure 3). Unmodified false-negative diagnoses of neoplasia included 21 T-cell lymphomas (14 mesenteric/intra-abdominal nodes in cats; 7 in a variety of nodes in dogs), 15 mast cell tumors, 10 metastatic carcinomas, 8 sarcomas, 2 melanomas, 2 B-cell lymphomas, and 1 histiocytic sarcoma. Modified false-negative diagnoses of neoplasia included 4 metastatic carcinomas, 2 B-cell lymphomas, and 1 T-cell lymphoma.

Twenty-six unmodified diagnoses of neoplasia were followed by modified phrases specifying the tumor type (e.g., metastatic carcinoma, consistent with anal sac apocrine adenocarcinoma) (Table 4). Twenty-five of 26 (96.1%) modified phrases specifying metastatic tumor type were from dogs, and 11/26 (42.3%) were from samples of the sublumbar lymph node (*P* < 0.0001). Probability terms accurately identified the specific tumor type in 22/26 (84.6%) cases.

**Table 4:** Accuracy of modified descriptions of metastatic tumor types in lymph node cytology samples from 25 dogs and 1 cat.

**Cytologic diagnosis**	**Modifier**	**Cytology tumor type**	**Histologic diagnosis**	**Concordant?**
Metastatic carcinoma	Most consistent	Thyroid carcinoma	Thyroid carcinoma	Yes
Metastatic carcinoma	Most consistent	ACA	Gastric ACA	Yes
Metastatic carcinoma	Most consistent	Apocrine gland ACA, anal sac	ACA of the anal gland	Yes
Metastatic carcinoma	Most consistent	Apocrine gland ACA, anal sac	ACA of the anal gland	Yes
Metastatic carcinoma	Most consistent	Apocrine gland ACA, anal sac	Anal sac carcinoma	Yes
Metastatic carcinoma	Most consistent	Apocrine gland ACA, anal sac	Anal sac ACA	Yes
Metastatic carcinoma	Probable	Apocrine gland ACA, anal sac	Carcinoma, anal gland	Yes
Metastatic carcinoma	Probable	Anal sac ACA	Anal sac gland carcinoma	Yes
Metastatic carcinoma	Probable	ACA	Anal sac gland ACA	Yes
Metastatic carcinoma	Probable	Anal sac carcinoma	ACA, presumed anal gland	Yes
Metastatic carcinoma	Consistent	Apocrine gland ACA, anal sac	Anal sac gland carcinoma	Yes
Metastatic carcinoma	Most likely	Transitional cell carcinoma	Transitional cell carcinoma	Yes
Metastatic carcinoma	Most suggestive	Apocrine gland ACA	Apocrine gland carcinoma	Yes
Metastatic ACA	Most consistent	Prostatic ACA	Presumptive transitional cell carcinoma^*^	Yes
Metastatic ACA	Compatible	Apocrine gland ACA	Apocrine gland carcinoma	Yes
Round cell tumor	Compatible	Histiocytic sarcoma	Presumed Langerhans cell histiocytosis	Yes
Round cell tumor	Probable	Histiocytic sarcoma	Histiocytic sarcoma	Yes
Metastatic neoplasia	Most consistent	Melanoma	Malignant melanoma	Yes
Metastatic neoplasia	Consistent	Melanoma	Presumptive amelanotic melanoma	Yes
Metastatic neoplasia	Probable	Melanoma	Malignant melanoma	Yes
Metastatic neoplasia	Very likely	Melanoma	Melanoma	Yes
Metastatic neoplasia	Possible	Melanoma	Malignant melanoma	Yes
Neoplasia	Probable	Lymphoma	Adenocarcinoma	**NO**^†^
Neoplasia	Probable	Plasma cell neoplasia	Sarcoma	**NO**^§^
Malignant neoplasia	Likely	Sarcoma	Transitional cell carcinoma	**NO**^¶^
Metastatic neoplasia (cat)	Suggestive of	Sarcoma	Squamous cell carcinoma	**NO**^¶^

*ACA indicates adenocarcinoma*.

* Biopsy report stated “suggestive of a transitional cell or prostatic epithelial origin.”

† *Cytology diagnosis was amended to “carcinoma” based on immunocytochemistry, 15 days prior to biopsy*.

§ *Cytology diagnosis was amended to “anaplastic sarcoma” based on immunocytochemistry, 10 days prior to biopsy*.

¶ *Extensive scirrhous/fibroblastic reaction noted in the biopsy specimen*.

**Figure 4:**
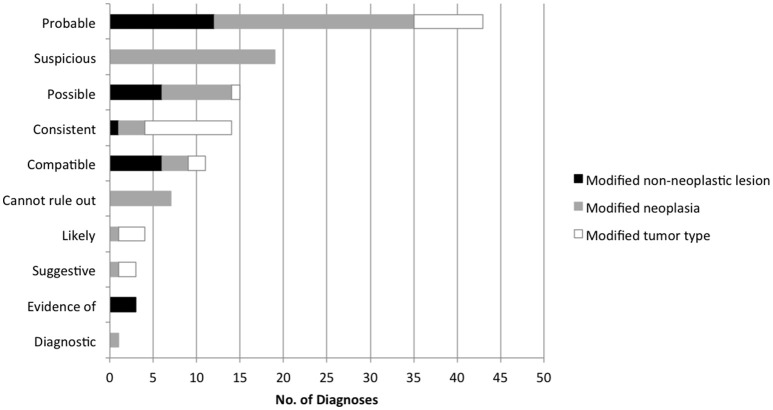
Frequency of individual terms used to express the probability of a diagnosis in lymph node cytology reports.

**Figure 5:**
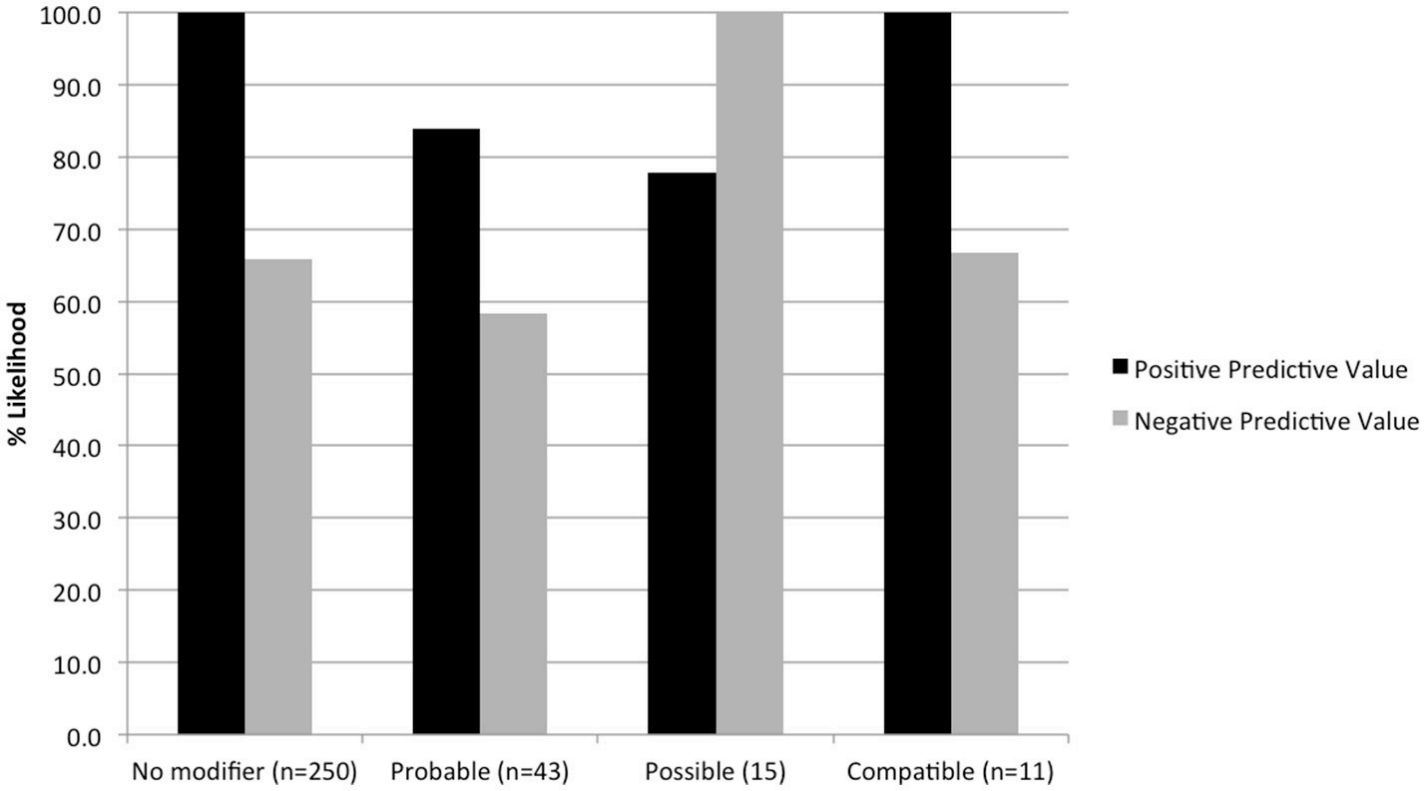
Positive and negative predictive values for terms used to modify both neoplastic and non-neoplastic diagnoses, based on histologic outcomes.

Ten unique probability terms were used to modify cytology diagnoses and specific tumor types (Figure 4). Terms that were further qualified by “most” (*n* = 13, i.e., “most consistent”), “highly” (*n* = 5, i.e., “highly suspicious”), or “very” (*n* = 1, i.e., “very likely”) were included in the category of the primary term for statistical analysis. “Consistent with” was more likely than other modifiers to be used in a secondary phrase referring to specific tumor type (*P* < 0.0001). “Cannot rule out” (or “cannot exclude”) was used in 7 cases to modify a diagnosis of lymphoma, following the diagnosis of a non-neoplastic lesion. Diagnostic accuracy for individual terms was as follows: probable (76.7%, *n* = 43), suspicious (73.7%, *n* = 19), possible (86.7%, *n* = 15), consistent with (85.7%, *n* = 14), compatible with (81.8%, *n* = 11), and cannot rule out (28.6%, *n* = 7). Diagnostic for, evidence of, likely, and suggestive were used 1-4 times each and all had 100% accuracy. No significant difference in accuracy was found between terms except for “cannot rule out” (*P* = 0.0368). Predictive values were calculated for those terms used most frequently to modify both benign and neoplastic diagnoses (Figure 5).

## Discussion

Based on confidence intervals and histologic outcomes, the PPV of a modified diagnosis of neoplasia was 60–83%, in contrast to 96–100% for unmodified diagnoses of neoplasia, and 12–49% for modified and unmodified benign diagnoses combined. In our large teaching hospital, probability terms were used to modify 25% of cytology diagnoses for lymph nodes from dogs and cats. Probability terms were used 3 times more often to modify a diagnosis of neoplasia compared with a non-neoplastic lesion, reflecting a greater level of uncertainty or caution in making a diagnosis of cancer. The frequency of use and accuracy rates of individual modifiers in this study were similar to those estimated previously by veterinary clinical pathologists and veterinary clinicians (3, 11). As in those studies, individual terms were poorly discriminatory. As such, a limited number of risk categories, based on the predictive values in this study, may be a more effective and accurate way to communicate the risk of malignancy than the wide range of subjective expressions currently used in lymph node cytology reports.

In a large survey of lymph node cytology samples submitted to a commercial diagnostic laboratory (19) the terms “possible” and “probable” were used by pathologists to modify 36.8% of neoplastic diagnoses and 11.6% of non-neoplastic diagnoses, similar to what was found in our study (40.5 and 12.2%, respectively). The slightly more frequent use of modifiers in our study may reflect the higher caseload of complex or difficult cases at a tertiary teaching hospital. Cytopathologists are understandably reluctant to over-interpret individual cells or unwilling to risk a false-positive diagnosis of neoplasia, as this could lead to unnecessary client concern, erroneous clinical decision-making, or euthanasia (2, 11). Our results indicate that probability modifiers are used appropriately to raise suspicion and increase the sensitivity of cytology (to 87%) and overall accuracy (to 78%) for detecting neoplasia, even though the diagnosis is less certain. The accuracy of modified diagnoses was even higher (85%) when applied solely to specific tumor types (where the diagnosis of neoplasia itself was certain); in these cases, accuracy increased even further (to 92%) when the results of ancillary immunocytochemical testing were taken into account. The higher level of confidence in identifying specific tumor type included more frequent use of “consistent with” and often involved sublumbar lymph nodes, suggesting the pathologist had prior knowledge or evidence of the primary tumor.

Although modified cytology diagnoses increased the sensitivity for detecting neoplasia, false-positive results also increased, resulting in decreased specificity. False positive diagnoses involved cases of lymphoma, metastatic melanoma, and mast cell neoplasia; 8 of 9 modified histologic diagnoses of neoplasia also were for these tumor types. Criteria established for the cytologic and histologic diagnosis of melanocytic and mast cell tumors have been proposed (17, 20, 21) but have not been widely validated and were not applied to all samples in this study. In some cases, excisional biopsy may be needed to fully assess metastasis of these tumors. Because of more false-positive results, modified cytologic diagnoses of neoplasia had a lower likelihood of cancer (PPV 73%) compared with unmodified diagnoses of neoplasia (PPV 100%), based on the histologic outcome. Notably, both false-positive diagnoses (of lymphoma and mast cell tumor) in cats were in mesenteric lymph nodes, which together with the high proportion of false-negative diagnoses in mesenteric nodes from cats emphasize the diagnostic challenge in evaluating cytology samples from this location.

False-negative diagnoses of neoplasia were almost always (90%) unmodified, indicating a lack of cytologic evidence to warrant suspicion of neoplasia. About one-third of these cases were T-cell lymphomas, most of which involved mesenteric lymph nodes in cats. T-cell lymphoma often is characterized by mature cell types (i.e., small cell lymphoma) that can be difficult to differentiate from reactive lymphoid hyperplasia, and examination of multiple tissue biopsies may be necessary to make a diagnosis (22). Suspicion or staging of alimentary lymphoma in cats warrants histopathologic examination. Difficulty in differentiating mastocytic inflammation from neoplasia, detecting focal metastases of carcinoma, and achieving exfoliation of mesenchymal neoplasms contributed to most other false negative diagnoses in this cohort. The likelihood of underlying neoplasia in lymph nodes with a non-neoplastic cytology result was similar for unmodified (34%) and modified (28%) diagnoses.

Because our study was limited to lymph nodes for which histologic examination was deemed necessary for staging or to confirm cytology results, the dataset had a slightly smaller proportion of cases of lymphoma, which is often diagnosed by cytology alone (18). This and other factors that affect the prevalence of lymphoma and metastatic neoplasia can affect predictive values when applied to different populations. However, the prevalence of neoplasia (41.9%) in the lymph node cytology study cited above (19) was similar to that for cytology samples in the present study (44.4%; 40% histologically confirmed). Further, the prevalence of metastatic neoplasia in canine and feline lymph nodes was 34.1% in another study (23); and the prevalence of neoplasia in a range of specimens examined by both cytology and histology was 49% (24). Thus, the predictive values obtained in our study are likely generalizable to other populations.

Another limitation of this study was the small sample size for individual terms, with some terms used too infrequently to draw conclusions about accuracy or predictive value. Preferential use of specific terms by individual (and multiple) pathologists likely contributed to variability in individual modifier use. Accuracy of individual terms in the present study was similar to that in previous surveys where clinical pathologists and veterinary practitioners assigned numeric values to probability terms and in which 10 of 18 descriptive terms (including the same terms used by clinical pathologists in the present study) were statistically indistinguishable (3, 11). This large variability among individual terms also was in concordance with studies conducted with medical pathologists, surgeons, trainees, and radiologists, both in delivering and receiving diagnoses (5–9). For the terms used most frequently, PPVs ranged from 74 to 92%, the exception being “cannot rule out,” which had a 28% likelihood of neoplasia, more consistent with a non-neoplastic lesion.

The results of this study provide the basis for establishing a few standard categories that convey the risk of malignancy in cytology specimens from lymph nodes, similar to what has been developed for specific tissues in medical cytopathology and in radiology (2, 12–16, 25, 26). Such categories, based on documented histologic outcomes, avoid the wide range of different (and ambiguous) probability terms that ultimately lead to a similar histologic outcome. Our results suggest the following categories may be appropriate: 96–100% (high risk, evidence of malignancy), 60–83% (suspicious for malignancy), and 12–49% (low risk, probably benign). An expanded description of the categories could include neoplasm-specific details, such as atypical mast cells, and the usual management or recommendations, such as biopsy in the case of well-differentiated lymphocytes in mesenteric nodes from cats. Just as for risk stratification categories in medical cytopathology, however, outcomes research is needed in veterinary cytopathology to implement and validate these categories.

In summary, probability modifiers as a group appropriately convey a greater amount of uncertainty in a diagnosis of neoplasia; while unmodified diagnoses of neoplasia have a near-certain likelihood of cancer. Cytologically benign lymph nodes have a lower (< 50%) but not negligible likelihood of underlying neoplasia, regardless of probability modifiers. These differential risk groups, based on histologically verified outcomes, provide a useful basis for establishing standard diagnostic categories that effectively and accurately convey the risk of malignancy in canine and feline lymph node cytology reports.

## Author contributions

C-KK collected the data, performed initial analyses, and drafted parts of the manuscript as part of her MS project. MC conceived of the study, developed the study design, refined and reanalyzed the data, and prepared the manuscript for publication.

### Conflict of interest statement

The authors declare that the research was conducted in the absence of any commercial or financial relationships that could be construed as a potential conflict of interest. MC is the Field Chief Editor of Frontiers in Veterinary Science; she was not involved in the peer review or decisions related to this manuscript.
